# Prevalence of Congenital Anomalies in Iran

**DOI:** 10.34172/aim.31287

**Published:** 2024-10-01

**Authors:** Ahmad Khaleghnejad-Tabari, Saeed Dastgiri, Hamid Soori, Alireza Ansari-Moghaddam, Haleh Ghaem, Mahmoud Latifi, Mohammad Reza Maracy, Saeed Aslanabadi, Fathollah Roshanzamir, Hamid Reza Forootan, Mehran Peivaste, Mehrdad Hoseinpour, Nasibeh Khaleghnejad-Tabari, Arameh Abbasian, Arghavan Haj-Sheykholeslami

**Affiliations:** ^1^Pediatric Surgery Research Center, Research Institute for Children’s Health, Shahid Beheshti University of Medical Sciences, Tehran, Iran; ^2^School of Medicine, Tabriz University of Medical Sciences, Tabriz, Iran; ^3^Faculty of Medicine, Cyprus International University, Nicosia, Republic of Cyprus; ^4^Health Promotion Research Center, Zahedan University of Medical Sciences, Zahedan, Iran; ^5^Shiraz University of Medical Sciences, Shiraz, Iran; ^6^Ahvaz University of Medical Sciences, Ahvaz, Iran; ^7^School of Public Health, Isfahan University of Medical Sciences, Isfahan, Iran; ^8^Isfahan University of Medical Sciences, Isfahan, Iran; ^9^Department of Community and Family Medicine, School of Medicine, Iran University of Medical Sciences, Tehran, Iran

**Keywords:** Birth defects, Congenital anomalies, Iran, Occurrence, Prevalence

## Abstract

**Background:**

Annually, 3-8 million infants are born with congenital anomalies worldwide, ranging from 3% to 7% of births in different countries. This study aimed to investigate the nationwide epidemiological features of birth defects in Iran.

**Methods:**

This cross-sectional study was conducted in six major regions across Iran. The data sources were the maternity facilities affiliated with regional universities of medical sciences. All children were examined by obstetricians, pediatricians, or midwives at birth, and newborns were followed until discharge from the facility for health status, maturity, and congenital defects.

**Results:**

A total of 138,643 births were registered in the maternity facilities across the study regions. Among these newborns, 3,458 cases were diagnosed with congenital anomalies, representing an overall prevalence rate of 249.4 per 10000 births (95% CI: 241.2-257.8). Genital organ anomalies exhibited the highest rates of defects in the country with 92.7 per 10000 births (95% CI: 86.4-98.9), followed by limb anomalies at 83.3 per 10000 births (95% CI: 77.4-89.3). The prevalence of respiratory system, chromosomal, and urinary tract anomalies was less than 10 per 10000 births.

**Conclusion:**

Until a national registry for congenital anomalies is established, this study provides essential data on the magnitude of the health problems caused by congenital anomalies in Iran. The findings would be vital for planning and evaluating antenatal screening for birth defects, particularly for high-risk groups and regions in the country.

## Introduction

 The term “Congenital Anomalies, or “Birth Defects” refers to structural abnormalities (e.g., cleft lip and palate), chromosomal disorders (e.g., Down syndrome), inherited diseases (e.g., cystic fibrosis), and inborn errors of metabolism (e.g., mitochondrial disorders and phenylketonuria) that are present at birth (live or stillborn). Congenital anomalies may be diagnosed before, during, or after birth later in life. They affect the structure or function of an organ or tissue in the body. In terms of clinical importance, congenital anomalies are typically categorized as minor or major defects.

 A complex interaction of genetic factors (i.e., gene mutations) and environmental factors (i.e., maternal metabolic or infectious diseases, drug use, and maternal smoking), consanguineous marriages, maternal age at childbirth, and socioeconomic factors play a significant role in birth defects. However, the causes remain largely unknown in many cases.

 Annually, 3-8 million infants are born with congenital anomalies worldwide, ranging from 3 to 7% of births in different countries.^1-4^ In Iran, systematic and evidence-based information on the occurrence of congenital disorders was scarce until the Tabriz Registry of Congenital Anomalies (TRoCA) was established in 2000. The primary objective of the TRoCA was to investigate the epidemiology of birth defects in the northwest of Iran.^5^ This registry reported an overall prevalence rate of 2.6% for newborns with at least one type of congenital anomaly at birth in the region. However, scattered studies indicated remarkable variations in the total prevalence and rates for specific types of defects across different parts of the country.^6,7^ The TRoCA program has estimated the occurrence of more than 100 000 cases of birth defects annually in the country in the absence of adequate preventive and screening programs.^1,2,8^ The current study aimed to investigate the nationwide epidemiological features of birth defects in Iran.

## Materials and Methods

 Iran, with a geographical area of 1.6 million square kilometers and an estimated population of 84.1 million, is located in the Middle East. The country is highly diverse, consisting of various religious and ethnic minorities, and is divided into 31 provinces. It also hosts one of the world’s largest refugee populations, mostly from Afghanistan in the east and Iraq in the west.^9^

 This cross-sectional investigation was conducted across six major regions of the country: north (Tehran province, including the capital city), northwest (East Azarbayjan), central area (Isfahan), southwest (Khoozestan), southeast (Sistan and Baluchestan), and south (Fras). The data sources were the maternity and pediatric facilities affiliated with the regional universities of medical sciences.

 All neonates born in these maternity facilities across the study area were examined at birth by gynecologists, obstetricians, neonatologists, pediatricians, or midwives. Newborns were followed until discharge from the facility for assessment of health status, maturity, and congenital defects. For each case, the basic demographic data and a complete clinical description of the birth defects were gathered using a pre-defined digital questionnaire. Anomalies were categorized according to the codes defined by the International Classification of Diseases (ICD-10), based on the primary diagnosis of the defects at birth under one of the main headings of congenital anomalies (Table 1).

**Table 1:** ICD-10 Codes of Congenital Anomalies

**Congenital Anomalies**	**Codes**
Selected groups	ICD-10 Codes
Respiratory system anomalies	Q30-Q34
Digestive system anomalies	Q38-Q45
Chromosomal anomalies	Q90-Q99
Genital organ anomalies	Q50-Q56
Head, neck, and face anomalies	Q10-Q18
Congenital heart diseases	Q20-Q28
Anomalies of limb	Q65-Q79
Urinary tract anomalies	Q60-Q64
Skin anomalies	Q82

*Note.* ICD-10: International classification of diseases.

 The sources for identifying congenital defects were hospital records and discharge forms. Fully trained and supervised data officers, including midwives, nurses, and medical coders, were assigned to collect the data for this study.

 The sample size was calculated using the population prevalence formula with the following indicators:

- Occurrence rate of 2.1% based on similar studies (to detect rare conditions),
- Confidence interval of 95%,
- Maximum acceptable error of 5.1% (d = 0.051),
- An estimated sample size of n = 307 267.

This sample size seemed excessively large in practice, making it infeasible to collect data from such a large number of subjects. Therefore, the investigators reduced the sample size due to financial and practical barriers identified in the pilot study (using 1% of the calculated sample size) conducted prior to implementing the main national investigation. Accordingly, the study sample, as determined in the pilot phase, comprised 138 736 births of Iranian origin, registered in maternity and pediatric facilities and hospitals in 2016. This reduced sample size made the study more manageable and practical. It was figured out that this sample size was sufficient to meet the study’s major objectives, as reported in this manuscript. However, for some rare conditions, the investigators acknowledged that the clustering effect should be considered when calculating the sample size, accounting for both the number of clusters and the number of births within each cluster to avoid underestimating the required sample size due to the clustering effect.

 Regarding the sampling method for this study, the proportional stratified random sampling method was used which involved dividing the population into provinces and then selecting cities based on their population weighting factors. Within each city, the birth centers were divided into three groups according to their frequency quartile, with one center selected from each group. A key characteristic of this sampling method is the use of clustering, where multiple births are selected from each cluster (city or birth center). This method involves selecting a group of births from a specific city or birth center rather than selecting individual births.

 The total prevalence rate was estimated by dividing the number of recorded cases of birth defects (numerator) by the total number of live and stillbirths (denominator). The 95% confidence intervals for the prevalence of congenital anomalies were calculated using the Poisson distribution, as recommended by the guidelines of the European Registry of Congenital Anomalies and Twins (EUROCAT).^3^ For inclusion in the data analysis, infants had to be born to a woman residing in the population area of the six major regions at the time of birth. An infant with more than one anomaly was counted only once in the numerator.

 This study was approved by the Ethics Committee of Shahid Beheshti University of Medical Sciences. The privacy and confidentiality of the children and families were strictly observed throughout data collection, analysis, access, and reporting.

## Results

 A total of 138 643 births (98.86% live births and 1.14% stillbirths) were registered in the maternity facilities across the study regions. Among these newborns, 3,458 cases of congenital anomalies were diagnosed, representing an overall prevalence rate of 249.4 per 10 000 births with a 95% confidence interval (CI) of 241.2-257.8.

Table 2 compares the basic characteristics of the study subjects, including maternal age at pregnancy, gestational age, parity, abortion history, birth weight, birth height, head circumference at birth, and gender of the neonates, between group 1 (healthy neonates) and group 2 (neonates with an anomaly at birth). Most cases were male, with 61.4% in group 1 and 50.2% in group 2. Except for maternal age at pregnancy and abortion history, significant differences were found between the two groups in gestational age, parity, birth weight, birth height, head circumference, and neonate gender.

**Table 2:** Basic Characteristics of Study Subjects

**Characteristics **	Group 1^*^				Group 2^**^			
	**Mean (SD)**	**95% CI**	**Median**	**Interquartile Range**	**Mean (SD)**	**95% CI**	**Median**	**Interquartile Range**
Mother age (y)	28.68 (5.96)	28.71-28.62	29	9	28.91 (6.02)	28.71-29.11	29	8
Gestational age (wk)	37.91 (2.89)	37.89-37.93	38	1	36.71 (5.07)	36.53-36.89	38	2
Parity	1.36 (1.41)	1.35-1.37	1	2	1.12 (1.33)	1.08-1.16	1	2
Abortion history	0.23 (0.60)	0.23-0.24	0	0	0.24 (0.59)	0.22-0.26	0	0
Weight at birth (g)	3114.14 (553.53)	3111.06-3117.22	3150	600	2966.09 (761.31)	2939.68-2992.51	3100	750
Height at Birth (cm)	34.29 (2.08)	34.28-34.31	34	2	34.09 (2.52)	33.99-34.18	34	2
Head circumference at birth (cm)	49.67 (3.08)	49.65-49.68	50	3	49.24 (3.65)	49.10-49.37	50	3
Neonate Gender	**%**				**%**			
Male	50.24				61.40			
Female	48.54				34.50			
Not recorded	1.22				4.10			

*Note.* SD: Standard deviation;CI:Confidence intervals. ^*^Healthy neonates; ^**^Neonates with an anomaly at birth.

**Figure 1:**
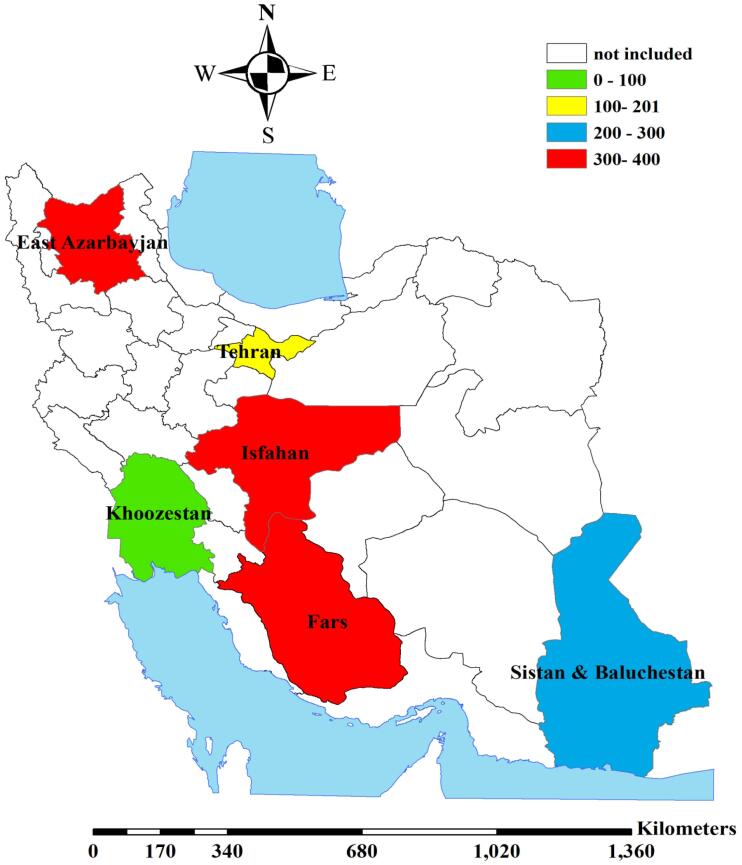
Total Prevalence (per 10 000 births) of Congenital Anomalies in Iran by Region

Table 3 and Figure 1 illustrate the total prevalence (per 10 000 births) of congenital anomalies across the study regions. The overall prevalence of birth disorders ranged from 94.3 per 10 000 births (95% CI: 83.3-106.4) in the southwest to 376.0 per 10 000 births (95% CI: 350.9-402.4) in the southern region, with almost similar figures in the central (326.0 per 10 000 births) and northwest (356.4 per 10 000 births) regions.

**Table 3:** Total Prevalence (Per 10 000 Births) of Congenital Anomalies in Iran by Region

**Regions**	**Total Births**	**Number of Anomalies**	**Prevalence**	**95% CI**
Tehran (capital city, north)	26 531	433	163.2	148.2-179.3
East Azarbayjan (northwest)	13 495	481	356.4	325.3-389.7
Isfahan (central)	27 334	891	326.0	304.9-348.1
Khoozestan (southwest)	28 093	265	94.3	83.3-106.4
Sistan and Baluchestan (southeast)	21 088	557	264.1	242.7-286.9
Fars (south)	22 102	831	376.0	350.9-402.4
Total	138 643	3458	249.4	241.2-257.8

*Note.* CI:Confidence intervals.

Table 4 presents the prevalence (per 10 000 births) of selected groups of congenital anomalies in Iran. Genital organ anomalies exhibited the highest rates of defects in the country, with a prevalence of 92.7 per 10 000 births (95% CI: 86.4-98.9), followed by limb anomalies at 83.3 per 10 000 births (95% CI: 77.4-89.3). The occurrence rates for the respiratory system, chromosomal disorders, and urinary tract anomalies were all below 10 per 10 000 births.

**Table 4:** Prevalence (Per 10 000 Births) of Selected Groups of Congenital Anomalies in Iran

**Selected groups **	**Prevalence**	**95% CI**
Respiratory system anomalies	8.8	6.9-10.8
Digestive system anomalies	13.1	10.7-15.4
Chromosomal anomalies	4.1	2.8-5.5
Genital organ anomalies	92.7	86.4-98.9
Head, neck and face anomalies	25.9	22.5-29.2
Congenital heart diseases	28.1	24.7-31.6
Anomalies of limb	83.3	77.4-89.3
Urinary tract anomalies	9.5	7.5-11.5
Skin anomalies	16.1	13.5-18.7

*Note.* CI:Confidence intervals.

## Discussion

 Cross-sectional studies on congenital disorders are mainly carried out to provide essential data for establishing baseline figures and identifying potential causes or clues to the etiology of birth defects and prevention at the population level.

 This epidemiological cross-sectional study was designed to estimate the prevalence and describe some features of congenital anomalies in Iran. The total prevalence of birth defects in Iran was estimated at 249.4 per 10 000 births. In comparison, the EUROCAT reported similar prevalence rates ranging from the lowest rate in Portugal (109.6 per 10 000 births) to the highest rates in Wales, the United Kingdom (368.2 per 10 000 births).^3^ In the northwest region of Iran, an overall prevalence of 262.9 (per 10 000 births) was previously reported by the TRoCA.^8^

 A study from Ethiopia reported a total prevalence of 62 per 10 000 births,^10^ while another study from the same country displayed a prevalence of 190 per 10 000 births.^11^ Another report from Nigeria in Africa presented varied prevalence rates between 280 per 10 000 births^12^ and 630 per 10 000 births.^13^ Given the lack of reliable evidence on the true figure of congenital anomalies in less developed countries, it is generally believed that the occurrence of birth disorders may be higher in these regions due to limited access to costly procedures needed for screening, diagnosis, and timely management of congenital anomalies. In addition, poor case ascertainment, data management methods, and possible underreporting should be taken into account when interpreting prevalence figures and making comparisons with low-income countries.

 Congenital heart defects, certain chromosomal disorders, and limb anomalies are among the most frequent types of congenital anomalies globally, while the anomalies of the ear, face, neck, and respiratory system tend to have lower prevalence rates. However, there are variations between countries and regions in the prevalence rates of specific groups of congenital defects.^2,3,14-22^ Interestingly, in our study, genital organ defects showed the highest rates of anomalies in the country.

 Although several studies have reported the impact of social and cultural factors, dietary habits, and familial susceptibility on the occurrence of birth disorders, the association between the occurrence of congenital anomalies and socio-demographic indicators, as well as ethnicity and cultural matters remains a challenge in the literature.^23-25^

 Similar to the findings of the current investigation, previously published reviews in Iran have demonstrated regional variations in the occurrence rates of congenital defects across the country.^6,7^ These variations can be attributed to several factors, including differences in the case ascertainment procedures, recording and coding of the defects, data sources, data collection methods, notification of fetal death, and the availability of professional personnel (e.g., obstetricians, gynecologists, neonatologists, pediatricians, nurses, and midwives, archives and medical documentation officers) in maternity and pediatric facilities across the study regions.

 Aside from the research published by the TRoCA on the occurrence and time pattern of congenital anomalies in northwestern Iran, the current study represents the first nationwide investigation into the prevalence of birth defects in Iran. However, we did not investigate the time pattern and thus could not report on downward or upward trends in specific categories of congenital anomalies over the years. Our study may have also underestimated the prevalence of congenital anomalies in the regions due to the lack of molecular and cytogenetic procedures, incomplete case ascertainment, and the unavailability of autopsies for stillbirths and neonatal deaths. Additionally, as this was a cross-sectional study, no longitudinal follow-up of the cases was conducted to investigate the prognosis of birth defects over age.

## Conclusion

 Until a national registry for congenital anomalies is established in Iran, this study provides essential data on the scale of health challenges caused by congenital anomalies in Iran. The findings of this investigation would be of importance for planning and evaluating antenatal screening for birth defects, particularly for high-risk groups and regions within the country.
